# Spatio-Temporal Patterns in *kdr* Frequency in Permethrin and DDT Resistant *Anopheles gambiae* s.s. from Uganda

**DOI:** 10.4269/ajtmh.2010.08-0668

**Published:** 2010-04

**Authors:** Katrijn Verhaeghen, Wim Van Bortel, Patricia Roelants, Paul Edward Okello, Ambrose Talisuna, Marc Coosemans

**Affiliations:** Department of Parasitology, Prince Leopold Institute of Tropical Medicine, Antwerp, Belgium; Epidemiological Surveillance Division, Ministry of Health, Kampala, Uganda; Department of Biomedical Sciences, Faculty of Pharmaceutical, Veterinary and Biomedical Sciences, University of Antwerp, Antwerp, Belgium

## Abstract

The planned upscaling of vector control strategies requires insight into the epidemiological consequences of vector resistance. Therefore, the pyrethroid and DDT resistance status of *Anopheles gambiae* s.l. was assessed in Uganda from 2004 to 2006, and spatial and seasonal variations in knockdown resistance (*kdr*) frequencies were analyzed in terms of epidemiological significance. *Anopheles gambiae* s.l. was DDT and pyrethroid resistant in central and eastern Uganda. The L1014S *kdr* allele frequencies varied from 3% to 48% in *An. gambiae* s.s. Although the homozygous resistant genotype was the most prevalent genotype among survivors, the genotypes could not entirely explain the bioassay results. In the dry season, the *kdr* frequency was significantly higher in *Plasmodium falciparum*-infected mosquitoes, indicating that mosquitoes bearing a *kdr* mutation have a better adult survival, hence a higher likelihood of becoming infectious. This study showed that *kdr* might have an epidemiological impact that could jeopardize the vector control strategies.

## Introduction

Uganda is one of the target countries benefiting from the United States President's Malaria Initiative (PMI), a 5-year initiative led by the United States Agency for International Development (USAID). The long-term goal of this PMI initiative is to cut malaria mortality by 50% in 15 African countries by 1) expanding access to long-lasting insecticide-treated bed nets (LNs) and indoor residual spraying (IRS), 2) promoting and supporting effective malaria treatment through the use of proven combination therapies, and 3) increasing prevention efforts targeted to pregnant women.^1^ The intensive use of insecticide in the malaria control activities means that widespread mosquito insecticide resistance could have a devastating effect on the planned upscaling of the vector control activities. In the African mosquitoes, insecticide resistance was first reported in *Anopheles gambiae* from the Ivory Coast^2^ and was soon found to be widespread in West, Central, and Eastern Africa.^3^

In Uganda, the malaria vector control is based on the use of bed nets impregnated with pyrethroid insecticides and the use of DDT for indoor residual spraying. Both insecticides target the voltage-gated sodium channel in the insect nervous system. In several insects, mutations in the *para*-type sodium channel gene have been linked to pyrethroid and DDT resistance, known as knockdown resistance (*kdr*).^4^ In *An. gambiae* s.s., knockdown resistance is caused by a single mutation resulting in a leucine-to-phenylalanine (L1014F, West African mutation) or leucine-to-serine (L1014S, East African mutation) change. Although both mutations are assumed to provide DDT and pyrethroid resistance, the L1014F *kdr* mutation might give a higher level of pyrethroid resistance than the L1014S *kdr* mutation.^5^,^6^ The impact of knockdown resistance on the efficacy of the vector control programs remains uncertain.^7^–^9^

Previous studies showed that both *kdr* mutations are present in *An. gambiae* s.s. of Uganda. The L1014S *kdr* mutation is present at a relative high frequency, whereas the L1014F *kdr* mutation is found at a low frequency in mosquitoes possessing the L1014S *kdr* mutation.^10^ The aim of this study is to assess the pyrethroid and DDT resistance status of the major malaria vectors and the role of the L1014S *kdr* mutation in providing pyrethroid and DDT resistance. Therefore, spatio-temporal patterns in *kdr* frequency were studied, the presence of the *kdr* mutation was assessed in sporozoite-infected and non-infected mosquitoes, and the presence of the L1014S *kdr* mutation was linked to the insecticide resistance status against pyrethroid and DDT as measured in the World Health Organization (WHO) standard susceptibility bioassay.

## Materials and Methods

### Mosquito collections.

**Figure 1.**
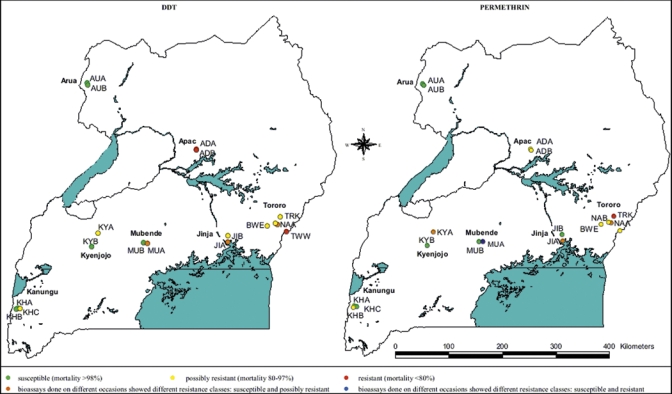
Mortality categories obtained for *Anopheles gambiae* s.l. in a WHO bioassay with DDT 4% and permethrin 0.75%.

In the framework of a study on the dynamics of malaria transmission (2001–2002), mosquitoes were collected indoors (from 8:00 pm to 6:00 am) and outdoors (from 8:00 pm to 11:00 pm) by human landing in seven villages, each located in a different district (Apac, Arua, Jinja, Kanungu, Kyenjojo, Mubende, and Tororo). The study locations were also sentinel sites for the monitoring of anti-malaria drug efficacy. For each village, 11 entomological surveys were conducted from June 2001 until May 2002 as described in Okello and others.^11^ From 2004 to 2006, mosquitoes were collected for the WHO bioassays using indoor human landing (8:00 pm to 6:00 am). Additional samples were trapped during the entire night by using a double net system: an outside untreated net with holes and an inside intact net with a sleeping person. At least two villages per sentinel district, including the villages surveyed during the longitudinal study, were prospected (Figure 1). All collected *Anopheles* mosquitoes were identified morphologically in the field using a simplified illustrated key adapted from Gillies and Coetzee.^12^

### DNA extraction.

One to six legs of individual mosquitoes were used for genomic DNA extraction, applying the procedure described in Collins and others.^13^ The DNA was resuspended in 25 µL TE buffer (10 mM Tris-HCl pH 8; 1 mM EDTA). A negative control was included with every set of extractions.

### Molecular identification.

The *Anopheles funestus* mosquitoes from the bioassays were identified following the protocol of Garros and others^14^ to assess the reliability of the morphological identification. Samples of the *An. gambiae* complex were identified using an adapted version of the polymerase chain reaction (PCR) developed by Scott and others^15^ to distinguish the different member species.^10^ Determination of the M and S molecular form was done, according to Favia and others,^16^ on the *An. gambiae* samples collected between 2001 and 2002.

### Bioassays.

Bioassays were performed on the morphologically identified *An. gambiae* s.l. and *An. funestus* mosquitoes collected from 2004 to 2006 using the standard WHO susceptibility test kit with discriminating concentrations of permethrin 0.75%, deltamethrin 0.05%, and DDT 4%.^17^ The impregnated and control papers were supplied by the Universiti Sains Malaysia and were not used more than five times. The exposure time was 60 min with tubes maintained in vertical position. Each test comprises replicates of 20 mosquitoes per test tube. The mosquitoes were kept under observation for 24 hours, supplied with 10% sugar solution, and the mortality was read after this 24-hour period. The bioassay results were summarized in three resistance classes as defined by WHO.^17^ A 24 hours post exposure mortality less than 80% indicates that the tested population is resistant, whereas a mortality higher than 98% indicates that the population is susceptible. With intermediate mortality levels the population is classified as suspected for resistance, and further confirmation is needed.^17^

### Detection of *kdr* mutations and analysis of *kdr* frequency.

The presence of the L1014S *kdr* mutation and the L1014F *kdr* mutation in *An. gambiae* s.s. was assessed on specimens collected during the longitudinal epidemiological study using adapted versions of the allele-specific PCRs (AS-PCR) developed by Martinez-Torres and others^5^ and described in Verhaeghen and others.^10^ A fluorescence resonance energy transfer/melt curve analysis (FRET/MCA)^10^ was used for quality control of the AS-PCR on a sample of the longitudinal survey specimens (*N* = 290). The FRET/MCA was used to detect the L1014S and L1014F *kdr* mutation in samples of the WHO bioassay (collected in 2004–2006). The *kdr* frequencies of the *An. gambiae* s.s. populations were analyzed using FSTAT.^18^ For one village (Tororo), seasonal variation in *kdr* frequency was analyzed on the samples of the longitudinal survey. The results obtained for survey 3, 7, and 8, representing the dry season, were pooled. The wet season grouping includes the pooled results of the remaining 8 surveys.^11^ Differences in *kdr* frequency between *Plasmodium falciparum* sporozoite-infected and non-infected mosquitoes were assessed. Previous results of the enzyme-linked immunosorbent assay (ELISA) tests^11^ to detect the circumsporozoite protein in head and thorax were used for that purpose. In addition, the entomological inoculation rate, as calculated by Okello and others,^11^ was reassessed for mosquitoes with (homozygous and heterozygous) and without (wild-type) the *kdr* mutation. The *kdr* genotype frequencies of mosquitoes exposed to the WHO bioassay were compared for dead and surviving mosquitoes using Genepop (version 3.4).^19^

### Detection of metabolic resistance.

The surviving control mosquitoes of the WHO bioassay (district Tororo: village NAA, NAB, and district Kanungu: village KHA) were stored in liquid nitrogen. Biochemical assays were performed on the *An. gambiae* control mosquitoes of Tororo (NAA, NAB; collected in 2004) to measure the involvement of metabolic resistance mechanisms. The levels of non-specific esterases, monooxygenase, and glutathione S-transferases (GST) were measured according to Penilla and others.^20^ Because no fully susceptible reference *An. gambiae* colony strain was available, the field collected *An. gambiae* s.s. from Kanungu (KHA in 2005) were used as controls in the bioassays and kept in liquid nitrogen to be used as reference. This reference population showed a 24 hour mortality of 100% against permethrin and 98% against DDT. The two-sample Kolmogorov-Smirnov Z test (SPSS version 15, SPSS Inc., Chicago, IL) was used to compare the enzyme levels.

## Results

### Molecular identification.

All mosquitoes of the longitudinal surveys (2001–2002), which were further used for *kdr* determination, were molecularly confirmed as *An. gambiae* s.s. Molecular identifications of *An. gambiae* s.s. mosquitoes (*N* = 904) indicate that only the S-form of *An. gambiae* is present in the seven districts.

For the bioassays, the molecular identifications of the *An. gambiae* s.l. (*N* = 1,435) and *An. funestus* (*N* = 44) mosquitoes confirmed the morphological identification (*An. gambiae* s.l.: 90%; *An. funestus*: 100% correctly identified). Only in Arua, 43% of the morphologically identified *An. gambiae* s.l. (*N* = 51) appeared to be *An. funestus*. No pyrethroid and DDT resistance was detected in this district; hence, the misidentification had no influence on the resistance data. In Jinja, 80% of the morphologically identified *An. gambiae* s.l. (*N* = 324) belonged to the *An*. *gambiae* s.l. complex, the remaining specimens showed an unknown ITS2 band (1,100 bp). When the bioassay mortalities were recalculated taking the molecular identification into account, the misidentification had no effect on the mortality classes obtained in the bioassays. *Anopheles gambiae* s.s. and *An. arabiensis* were sympatric in Tororo (5% *An. arabiensis*) and Jinja (1% *An. arabiensis*). *Anopheles arabiensis* was found among bioassay survivors and non-survivors.

### WHO bioassays (2004–2006).

All bioassays were done on at least 75 mosquitoes. In the northwestern part of the country, no pyrethroid or DDT resistance has been detected so far. The DDT resistance is mainly found in the *An. gambiae* s.l. populations of the central and eastern parts of the country. Permethrin resistance was observed in the districts of Tororo and Apac. The permethrin resistant status of *An. gambiae* s.s. in Mubende needs some clarification, because bioassays done at different occasions showed conflicting results (Figure 1, Table 1). The resistance status of *An. gambiae* s.l. against deltamethrin was assessed in *An. gambiae* s.l. of the districts Jinja, Kanungu, and Tororo. Only in Tororo, the *An. gambiae* s.l. population was classified as suspected resistance against deltamethrin. In Apac, the *An. funestus* populations showed suspected resistance against DDT and permethrin (Table 1).

### Presence of *kdr* mutations.

For 2001–2002, substantial differences in L1014S *kdr* frequency were found among the seven sentinel sites (*F*_st_ = 0.083, *P* = 0.001). The *kdr* frequencies did not differ (*P* > 0.05) among the sites of Mubende, Kanungu, Kyenjojo, Apac, and Tororo and fluctuated between 25% and 30%. The peri-urban village of Jinja had an intermediate *kdr* frequency of 13%, whereas the lowest value was found in Arua (3%). The L1014S *kdr* frequency of Jinja and Arua differed significantly from each other (*P* < 0.0005) and from the five other sites (*P* < 0.002; except Jinja versus Kenjojo *P* = 0.019). The L1014F *kdr* allele was only found in *An. gambiae* s.s. mosquitoes, which already possessed the L1014S *kdr* allele and occurred only in Tororo (0.3%) and Mubende (3%) at a low frequency (Table 2). The quality control done with the FRET/MCA, showed for the L1014S and L1014F *kdr* mutation discrepancy for 10 and 2 *An. gambiae* s.s. specimens, respectively (*N* = 290). The discrepancies between the AS-PCR and the FRET/MCA had no effect on the *kdr* frequencies measured in the different *An. gambiae* s.s. populations.

During 2004–2006, the L1014S *kdr* frequency of Kanungu differed significantly from Mubende and Tororo (*P* = 0.005), whereas the L1014S *kdr* frequency of Kyenjojo differed from Tororo (*P* = 0.005) (Table 2). Between 2001 and 2002 and 2004 and 2006 there were significant increases in L1014S *kdr* frequency for the districts Jinja, Mubende, and Tororo (*P* < 0.002). Even during 2001–2002, temporal variations in L1014S *kdr* frequencies were found among the different surveys (monthly interval) for the districts Tororo (*F*_st_ = 0.010, *P* < 0.002, range 24–43%) and Jinja (*F*_st_ = 0.057, *P* = 0.020, range 2–27%). Seasonal variation in L1014S *kdr* frequency was further examined on the *An. gambiae* s.s. mosquitoes collected between 2001 and 2002 in Tororo.

**Figure 2.**
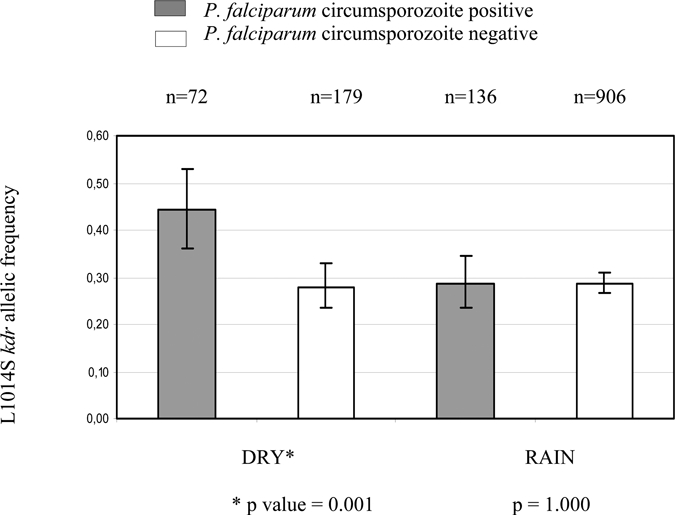
Impact of the season and the *Plasmodium falciparum* circumsporozoite status on the L1014S *kdr* allelic frequency in Tororo detected during the 1-year entomological study (2001–2002).

During the period 2001–2002, no differences in L1014S *kdr* allelic frequency were found between the dry and wet season (*F*_st_ = 0.003; *P* = 0.0858). Comparison of positive and negative mosquitoes for circumsporozoite protein detection by ELISA showed that mosquitoes that were positive for *P. falciparum* sporozoites had a higher L1014S *kdr* allelic frequency (*F*_st_ = 0.006; *P* = 0.033). When both data on season and the sporozoite status were combined, the difference in *kdr* allelic frequency was even more pronounced. In the dry season, the L1014S *kdr* allelic frequency was significantly higher in mosquitoes that contained *P. falciparum* sporozoites compared with non-infected mosquitoes (*F*_st_ = 0.055; *P* = 0.001). In the wet season, the L1014S *kdr* frequency was found to be similar in sporozoite positive and negative mosquitoes (*F*_st_ = −0.002; *P* = 1.000) (Figure 2).

In Tororo, the entomological inoculation rate (EIR), which estimates the level of exposure to malaria parasite-infected mosquitoes was also measured for the different surveys. It showed that both, mosquitoes with and without the L1014S *kdr* mutation, contribute to the malaria transmission. Moreover, mosquitoes with and without *kdr* contributed equally to the annual entomological inoculation rate (AEIR): the AEIR for non *kdr* mosquitoes was 222 bites per man-year versus 234 bites per man-year for L1014S *kdr* positive mosquitoes. However, in the dry season, mosquitoes bearing the *kdr* mutation contribute 70% to the EIR.

### Role of L1014S *kdr* mutation in providing resistance.

**Figure 3.**
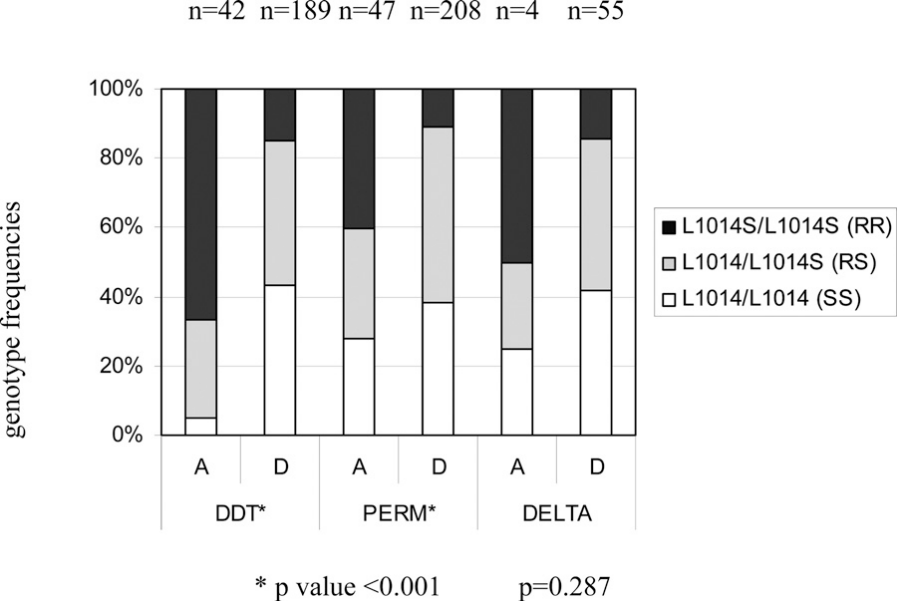
Pooled *kdr* genotypes frequencies found in live (A) and dead (D) *Anopheles gambiae* s.s individuals (collected from 2004 to 2006). The *P* values found between surviving and dead *Anopheles* species are shown at the bottom.

To assess the role of the L1014S *kdr* allele in conferring pyrethroid and DDT resistance in *An*. *gambiae* s.s., the *kdr* genotype was determined for dead and alive mosquitoes detected in the WHO bioassay (Figure 3). Among both bioassay survivors and non-survivors, all *kdr* genotypes (L1014/L1014, L1014S/L1014, and L1014S/L1014S) were found. Yet, the homozygous resistant genotype (L1014S/L1014S) was the most prevalent genotype among the bioassay survivors. This correlation was found for both DDT and permethrin, but was stronger for DDT (*F*_st_ = 0,317 for DDT; *F*_st_ = 0,074 for permethrin). For deltamethrin, there were no differences in genotypic distribution, although this can be caused by the limited number of specimens surviving the deltamethrin exposure (Figure 3).

Although there was a significant *kdr* genotype differentiation between bioassay survivors and non-survivors, homozygous susceptible mosquitoes were found among bioassay survivors, which can suggest that other resistance mechanisms are present. To assess the involvement of metabolic resistance mechanisms, biochemical assays were performed on the resistant *An. gambiae* s.s. population of the Tororo district (NAA and NAB). A significant increase in esterase activity was measured with the substrate para-nitrophenyl acetate (PNPA) in the resistant *An. gambiae* s.s. populations of Tororo, compared with activity measured for the KHA population. The levels of monooxygenases and GST activities measured in the *An. gambiae* s.s. populations of NAA and NAB did not differ from the levels measured in KHA (Table 3).

## Discussion

In the Roll Back Malaria Initiative, malaria vector control programs have a prominent place.^1^ Information on the resistance status of the vector can be used to guide the insecticide use for vector control programs. Therefore, WHO bioassays were used in seven sentinel sites throughout Uganda to assess the insecticide resistance status of the main malaria vectors *An. gambiae* s.l. and *An. funestus* against permethrin, deltamethrin, and DDT.

The DDT resistance in *An. gambiae* s.l. was found to be widespread in Uganda and mainly observed in the central and eastern parts of the country. Permethrin resistance was mainly observed in the densely populated eastern part of Uganda (Tororo and Apac) where cotton was grown in the past. In Tororo, the *An. gambiae* s.l. population was classified as suspected resistance against deltamethrin. The permethrin resistance status of *An. gambiae* s.l. in Mubende still needs clarification. In Apac, *An. funestus* express some evidence of DDT and permethrin resistance.

Clear spatial differences in *kdr* allele frequencies were observed in Uganda. In many sites, the *kdr* allele frequency rose above 30%, which is much higher than the 8% observed in nearby Kenya.^21^ In addition to the spatial patterns in *kdr* frequency; temporal variations were observed in Tororo and Jinja between the different study periods 2001–2002 and 2004–2006. These spatio-temporal patterns in *kdr* allelic frequencies were generated by a complex interplay between the population biology and genetics of the vector, the insecticide pressure presence in the ecosystem, the role the mutation plays in conferring resistance and its associated fitness cost.

In general, the lowest L1014S *kdr* frequencies were detected in the permethrin and DDT susceptible *An. gambiae* s.s. populations of western Uganda, areas where coffee, tea, and tobacco plantations are present. The highest L1014S *kdr* frequencies occurred in cotton-growing areas of Uganda. Furthermore, in *An*. *gambiae* s.s. populations of Burkina Faso, the L1014F *kdr* frequency was high in cotton-growing and urban areas and low in areas with limited insecticide selection pressure from agriculture (e.g., rice fields).^22^ The monthly variation in *kdr* allelic frequencies observed during the longitudinal study (2001–2002) ranged from 24% to 43% in Tororo and from 2% to 27% in Jinja, which raised the question of whether this variation can be caused by seasonal variation in insecticide pressure. However, when data were grouped by season, no variation in *kdr* allelic frequency was observed. This contrasts with the situation in southwestern Nigeria where the L1014F *kdr* frequency in *An. gambiae* s.s. in the dry season was significantly lower than in the wet season.^23^ Yet, when the *P. falciparum* sporozoite status is taken into account, the *kdr* frequency was significantly higher in *P. falciparum*-infected mosquitoes during the dry season. This might indicate that mosquitoes bearing a *kdr* have a fitness advantage in the dry season, e.g., by seasonal changes in insecticide pressure, advantage resulting in better adult survival, hence in a higher likelihood of becoming infectious. This study shows that mosquitoes bearing *kdr* mutations contributed 70% to the malaria transmission in the dry season. Because *kdr* frequencies can increase rapidly,^24^ this longevity advantage might have enormous implications for the malaria transmission and might jeopardize the current resistance management strategies. Previous studies on the fitness cost of resistance mechanism^25^ concluded that metabolic resistance mechanisms often lead to serious disadvantages, whereas *kdr*-type mechanisms of pyrethroid resistance may confer little or no effect on overall fitness. If fitness cost of *kdr* is low, the *kdr* frequency can remain high, even in the absence of insecticide pressure. In addition, the incomplete recessive nature of the *kdr* mutation^26^ will keep it protected from selection in the heterozygous state.

Moreover, the role of L1014S *kdr* mutation in conferring insecticide resistance was assessed. Previous studies on the role of the L1014S *kdr* mutation assumed that this *kdr* mutation in *Culex pipiens* and *An. gambiae* s.s. confers high levels of DDT resistance and low levels of permethrin resistance.^6^,^26^ The WHO bioassays performed on *An. gambiae* s.s. from Uganda showed that the homozygous resistant genotype (L1014S/L1014S) is the most prevalent genotype among the bioassay survivors. This correlation was found for both DDT and permethrin, but was stronger for DDT. However, the *kdr* genotypes do not explain entirely the bioassay results. The L1014S *kdr* frequency has already reached 20% in the DDT susceptible *An*. *gambiae* s.s. populations of Kanungu, showing that the mutation is not absolute in conferring phenotypic insecticide resistance. The high proportion of *kdr* homozygous susceptible specimens, which survived the WHO bioassays, may suggest the implication of other insecticide resistance mechanisms. The biochemical assays showed a significant increased esterase activity in pyrethroid and DDT resistant *An. gambiae* s.s. populations of Tororo (NAA, NAB) compared with pyrethroid and DDT susceptible population of Kanungu (KHA). Synergist studies should be performed on *An. gambiae* s.s. of Tororo to identify the detoxifying role of the esterases, but it is likely that these esterases confer pyrethroid resistance as in the neighboring country, Kenya, both esterases and monooxygenases are involved in pyrethroid resistance in *An. gambiae* s.s.^27^

As different pyrethroid and DDT resistance levels and resistance mechanisms were observed in the main vectors of Uganda, the impact of the detected insecticide resistance on the current vector control programs should be further determined by performing experimental hut studies. Previous studies have shown that a high L1014F *kdr* frequency in *An. gambiae* s.s. populations of the Ivory Coast had no effect on the effectiveness of pyrethroid-treated nets.^7^,^28^–^30^ However, in neighboring countries (Benin and Equatorial Guinea) insecticide-treated nets (ITN) or IRS failed to control *kdr* resistant *An. gambiae*.^9^,^31^ Metabolic pyrethroid resistance in *An. funestus* of South Africa required a switch back from pyrethroid insecticides to DDT for house spraying to restore the malaria control,^32^ whereas metabolic resistance in *An. gambiae* s.s. of Cameroon did not influence the personal protection afforded by ITNs by sustained irritancy, but did reduce the vector mortality, which could in turn limit the mass effect.^33^ However, the presence of multiple resistance mechanisms in the malaria vectors of Uganda might influence the planned upscaling of IRS and LNs. Resistance management strategies could be implemented to delay the emergence of insecticide resistance. Pyrethroids could be preserved for ITNs, whereas non-pyrethroid insecticides such as carbamates or organophosphates could be used for IRS. In addition, WHO bioassays should be performed regularly to monitor trends in insecticide resistance as net bioassays performed in western Uganda have showed the dynamic properties of pyrethroid resistance by observing a 1.5-fold decrease in pyrethroid mortality in *An. gambiae* s.l. over a period of only 10 years.^34^ The presence, though at small frequency, of the West African *kdr* mutation needs to be carefully followed, as this *kdr* mutation was previously related with high levels of both pyrethroid and DDT resistance.^5^,^35^,^36^

## Figures and Tables

**Table 1:** Twenty-four hours mortality observed in the WHO bioassay with discriminating concentrations of DDT, permethrin, and deltamethrin (mortality was not corrected for mortality in the controls)*

Species	District	Village	Month/Year	24-hrs mortality in WHO bioassays		
				4% DDT (n)	0.75% Permethrin (n)	0.05% Deltamethrin (n)
*An. funestus*	Apac	ADA	Jan 2005	99 (101)	99 (97)	Nd
			Sept 2006	100 (80)	93 (80)	Nd
		ADB	Oct 2004	95 (177)	98 (175)	Nd
			Jan 2005	97 (97)	98 (138)	Nd
			Sept 2006	81 (80)	92 (75)‡	Nd
*An. gambiae*	Apac	ADA	Sept 2006	76 (80)	81 (80)	Nd
		ADB	Sept 2006	63 (80)	80 (80)	Nd
	Arua	AUA	April 2005	99 (200)	100 (193)	Nd
		AUB	April 2005	100 (202)	100 (199)	Nd
	Jinja	JIA	Jan 2005	81 (80)	96 (79)	Nd
			Nov 2006	67 (87)	74 (85)	98 (93)
		JIB	July 2004	87 (202)	99 (195)†	100 (77)
	Kanungu	KHA	Oct 2004	97 (180)‡	Nd	100 (98)
			May 2005	97 (197)	100 (198)	Nd
		KHB	May 2005	100 (201)	88 (191)†	Nd
			Aug 2006	99 (78)	95 (80)	Nd
		KHC	Oct 2006	97 (91)	99 (83)	100 (97)
	Kyenjojo	KYA	May 2005	93 (101)	95 (80)	Nd
			Dec 2006	81 (85)	61 (85)	100 (85)
		KYB	May 2005	98 (80)	100 (75)	Nd
	Mubende	MUA	April 2005	86 (88)	99 (81)	Nd
			Dec 2006	72 (85)	54 (85)	87 (85)
		MUB	April 2005	97 (79)	100 (99)	Nd
	Tororo	NAA	June 2004	93 (204)	76 (200)†	98 (97)
			Feb 2006	86 (101)	91 (91)	100 (80)
			Nov 2006	70 (200)	85 (199)	97 (97)
		NAB	June 2004	90 (214)§	84 (215)§	95 (81)
		BWE	Aug 2006	83 (90)	96 (92)	100 (100)
		TRK	March 2006	93 (151)	78 (166)	98 (178)
		TWW	March 2006	60 (161)	81 (151)	97 (178)

* Nd = not done.

Mortality in the controls was always < 5% except † = 5%; ‡ = 6%; § = 7%.

**Table 2:** L1014S and L1014F *kdr* frequencies observed in *Anopheles gambiae* s.s. populations of the several districts of Uganda (number between brackets)*

District	L1014S *kdr* frequency				L1014F *kdr* frequency			
	2001–2002		2004–2006		2001–2002		2004–2006	
Apac	29%	(777)	na		0%	(175)	Na	
Arua	3%	(758)	na		0%	(55)	Na	
Jinja	13%	(111)	34%	(61)	0%	(172)	0%	(61)
Kanungu	27%	(495)	20%	(46)	0%	(78)	0%	(46)
Kyenjojo	31%	(62)	32%	(152)	0%	(10)	0%	(152)
Mubende	25%	(173)	48%	(106)	3%	(18)	0%	(106)
Tororo	29%	(1293)	47%	(399)	0.3%	(520)	0%	(399)

* Period 2001–2002: refers to the longitudinal study.^11^ Period 2004–2006: bioassays. na = not available.

**Table 3:** Mean and standard error (SE) obtained for the esterase, monooxygenase, and glutathione S-transferases (GST) assay on *Anopheles gambiae* s.s. populations collected from 2004 to 2006*

Site	WHO bioassay†			Biochemical assays									
	DDT4%	Permethrin 0.75%	Deltamethrin 0.05%	n	Esterase (PNPA)(µmol/min/mg protein)			Monooxygenase(nmol equivalent unit cyt P450 /mg protein)			GST (CDNB)(mmol/min/mg protein)		
					Mean	SE	*P* value‡	Mean	SE	*P* value‡	Mean	SE	*P* value‡
KHA	S	S	S	30	0.0403	0.0030		0.2161	0.0280		0.0645	0.0183	
NAA	SR	R	S	59	0.0905	0.0079	↑**0.000**	0.1565	0.0194	0.090	0.2049	0.0560	0.366
NAB	SR	SR	SR	28	0.0846	0.0141	≠**0.018**	0.1898	0.0531	0.100	0.0613	0.0151	0.791

* The esterase activity was measured with para-nitrophenyl acetate (PNPA), whereas the GST activity was measured with 1-chloro-2,4-dinitrobenzene (CDNB) as substrate.

† Mortality categories: S = susceptible (mortality > 98%); SR = suspected resistance (mortality between 80% and 98%); R = resistant (mortality < 80%).

‡ ↑Mean rank of population is increased compared with the mean rank of the KHA *An. gambiae* s.s. population.
